# Finger Allergic Contact Dermatitis by Proxy Induced by Feline Thiamazole Cream

**DOI:** 10.1111/cod.70216

**Published:** 2026-07-30

**Authors:** Gabriela Blanchard, Ophélie Marchal, Olivier Sorg, Emmanuel Laffitte, Pierre Piletta‐Zanin

**Affiliations:** ^1^ Department of Dermatology and Venereology University Hospital of Geneva Geneva Switzerland; ^2^ Clinical Pharmacology and Toxicology Unit University of Geneva Geneva Switzerland

**Keywords:** allergic contact dermatitis, CAS no. 60‐56‐0, case report, methimazole, thiamazole

Thiamazole (also called methimazole, CAS no. 60‐56‐0) is a potent peroxidase inhibitor used to treat hyperthyroidism [1]. Transdermal application of thiamazole has been used in hyperthyroid cats as an alternative to oral therapy [2].

## 1 Case Report

We present the case of a 50‐year‐old female who developed localised erythematous, scaly plaques with fissures on the right index and middle fingers, consistent with eczema (Figure 1A,B). No inflammatory lesions were present elsewhere on the body. The patient was right‐handed. Further history revealed that, for the last 6 years, she had been using these fingers to apply a thiamazole‐containing cream to the ears of her hyperthyroid cat. The cat did not exhibit any cutaneous signs suggestive of allergic contact dermatitis. A Repeated Open Application Test with the thiamazole‐containing cream was positive (Figure 1C). Patch tests were performed using the European baseline, preservative and emulsifier series (supplied by Chemotechnique Diagnostics, Vellinge, Sweden) using IQ Ultra chambers (Chemotechnique Diagnostics) and the thiamazole‐containing cream, including its individual ingredients, kindly provided by the manufacturer. Readings performed on Day (D) 2 and D4 according to the International Contact Dermatitis Research Group (ICDRG) criteria showed a single positive reaction to thiamazole (thiamazole pure and thiamazole 20% in petrolatum: D2++, D4+++; thiamazole 20% in alcohol: D2+, D4++) (Figure 1D). Five unexposed controls were negative to thiamazole 20% in petrolatum. Avoidance of direct contact with the thiamazole‐containing cream with the application of topical clobetasol led to a rapid and complete clinical remission.

**FIGURE 1:**
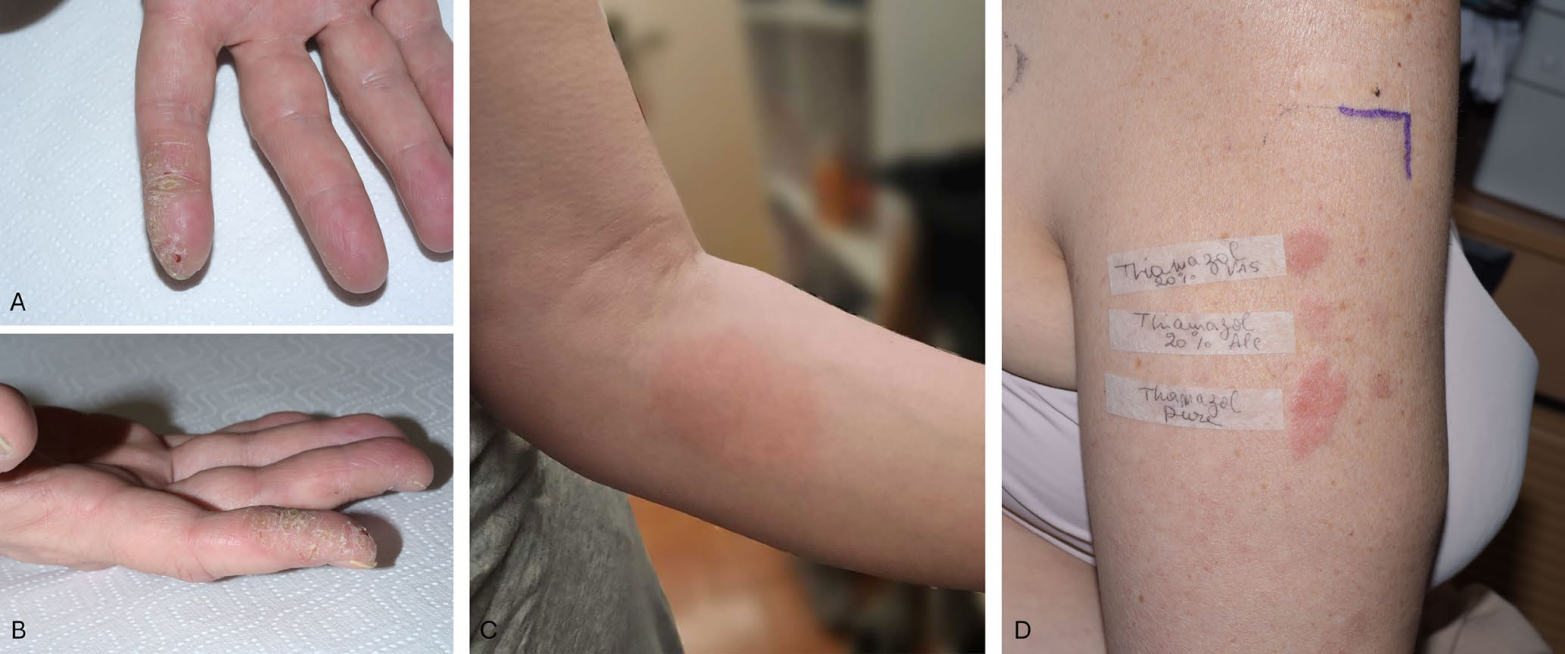
(A, B) Clinical presentation of allergic contact dermatitis caused by thiamazole. (C) Positive Repeated Open Application Test with the thiamazole‐containing cream. (D) Patch testing showing a strong positive (+++) reaction to thiamazole on Day 4.

## 2 Discussion

To the best of our knowledge, we describe the first case of allergic contact dermatitis caused by thiamazole. In such cases, avoidance of thiamazole, as well as the remaining thionamide antithyroid drugs (thiamazole prodrug carbimazole and propylthiouracil), should be recommended due to potential class cross‐reactivity and the risk of systemic contact dermatitis [3]. Cases of thiamazole‐induced vasculitis [4], cutaneous lupus erythematosus [5], erythema nodosum [6] and maculopapular exanthema [7] have been reported following systemic use of thiamazole. Topical thiamazole has been increasingly used not only in veterinary medicine, but also as an emerging depigmenting agent [8, 9]. Allergic contact dermatitis by proxy, also known as consort or connubial allergic contact dermatitis, is an uncommon form of allergic contact dermatitis resulting from indirect exposure to an allergen carried by another individual [10] or, rarely, by an animal. Pets may act as vectors for allergens such as plant resins, topical veterinary medications or grooming products. The use of protective gloves when applying topical medications to animals is recommended to minimize inadvertent allergen transfer and sensitization.

## Author Contributions

**Gabriela Blanchard:** conceptualization, investigation, writing – original draft, methodology, validation, writing – review and editing. **Ophélie Marchal:** writing – review and editing. **Olivier Sorg:** writing – review and editing. **Emmanuel Laffitte:** conceptualization, investigation, methodology, writing – review and editing, validation. **Pierre Piletta‐Zanin:** conceptualization, investigation, methodology, validation, writing – review and editing.

## Consent

The authors obtained consent from the patient for their photographs and medical information to be published in print and online and with the understanding that this information may be publicly available. Patient consent forms were not provided to the journal but are retained by the authors.

## Conflicts of Interest

The authors declare no conflicts of interest.

## Data Availability

The data that support the findings of this study are available from the corresponding author upon reasonable request.
